# The Second Round of a Population‐Based Seroprevalence Study of Anti–SARS‐CoV‐2 Antibodies and COVID‐19 Vaccination Assessment in the Republika Srpska, Bosnia and Herzegovina

**DOI:** 10.1111/irv.70053

**Published:** 2025-01-16

**Authors:** Jela Aćimović, Biljana Mijović, Srđan Mašić, Miroslav Petković, Dragana Puhalo Sladoje, Darija Knežević, Jelena Đaković Dević, Dragan Spaić, Jelena Vladičić‐Mašić, Dejan Bokonjić, Mirza Palo, Aisling Vaughan, Richard Pebody, Anthony Nardone, Ranko Škrbić

**Affiliations:** ^1^ Department of Epidemiology Public Health Institute of the Republika Srpska Banja Luka Republika Srpska Bosnia and Herzegovina; ^2^ Department of Epidemiology, Faculty of Medicine University of Banja Luka Banja Luka Republika Srpska Bosnia and Herzegovina; ^3^ Department of Primary Health Care and Public Health, Faculty of Medicine University of East Sarajevo Foča Republika Srpska Bosnia and Herzegovina; ^4^ Regional Centre Foča Public Health Institute of the Republika Srpska Foča Republika Srpska Bosnia and Herzegovina; ^5^ Centre for Biomedical Research, Faculty of Medicine University of Banja Luka Banja Luka Republika Srpska Bosnia and Herzegovina; ^6^ Centre for Biomedical Research, Faculty of Medicine University of East Sarajevo Foča Republika Srpska Bosnia and Herzegovina; ^7^ Department of Internal Medicine, Faculty of Medicine University of East Sarajevo Foča Republika Srpska Bosnia and Herzegovina; ^8^ Department of Pediatrics, Faculty of Medicine University of East Sarajevo Foča Republika Srpska Bosnia and Herzegovina; ^9^ Health Emergency Programme World Health Organization Regional Office for Europe Copenhagen Denmark; ^10^ WHO Country Office for Bosnia and Herzegovina Sarajevo, Bosnia and Herzegovina; ^11^ Department of Epidemiology Epiconcept Paris France

**Keywords:** Bosnia and Herzegovina, COVID‐19, population‐based study, SARS‐CoV‐2 antibodies, SEROPREV, seroprevalence, UNITY

## Abstract

**Introduction:**

The aim of the study was to assess the seroprevalence of SARS‐CoV‐2 in the Republika Srpska, Bosnia and Herzegovina, after five waves of COVID‐19 and 1 year after introduction of vaccination to better understand the true extent of the COVID‐19 pandemic in the population of the Republika Srpska and role of vaccination in achieving herd immunity.

**Methods:**

The population‐based study was conducted from December 2021 to February 2022 in a group of 4463 individuals in the Republika Srpska. Total anti–SARS‐CoV‐2 antibodies were determined in serum specimens using the Wantai total antibody ELISA assay. Quantitative analysis, using Kantaro IgG assays, was performed in subsamples (1273 specimens) to asses and compare levels of IgG among vaccinated, recovered and participants with hybrid immunity. To adjust for age and gender distribution in sample, poststratification method is applied.

**Results:**

The overall cumulative seroprevalence was 94.6% (95% CI = 93.9–95.3). Significantly higher seroprevalence rates were observed among vaccinated 97.8% (95% CI = 97.3–98.4) comparing to unvaccinated participants (89.5%, 95% CI = 88.0–91.0). Seroprevalence increases with the number of received doses. Among various professions, the highest seroprevalence was found in the service industry (98.1%), education (98.0%) and healthcare (96.9%). We found that 2.2% of vaccinated and 3.6% of participants with SARS‐CoV‐2 positivity during 2021 had no detectable IgG antibodies. Both seroprevalence (98.6%) and antibody titres (1094.4 AU/mL) were significantly higher among people with hybrid immunity.

**Conclusion:**

Our findings reveal a 2.3‐fold increase in seroprevalence of SARS‐CoV‐2 antibodies due to infection and vaccination, comparing to the first study performed 1 year earlier. This study provides better understanding of the SARS‐CoV‐2 transmission and highlights the important role of the vaccination in achieving the population immunity. Periodically conducted population‐based seroprevalence studies are important to understand temporal trends and assess surveillance system performance and public compliance with vaccination policies.

## 1 Introduction

Since the beginning of the COVID‐19 pandemic, more than 768 million cases were reported globally, with more than 6.9 million deaths [1]. In the Republika Srpska, Bosnia and Herzegovina, from 5 March 2020, when the first case of COVID‐19 was confirmed, to the end of February 2022, a total of 111,247 COVID‐19 cases were detected, during five epidemic waves. A total of 414,478 *reverse transcription polymerase chain reaction* (RT‐PCR) tests were performed with overall test positivity of 26.8%. As of 28 February 2022, the cumulative incidence rate, as total number of PCR confirmed cases of SARS‐CoV‐2 infections per population of 100.000, was 9691. During the same period, a total of 6281 death cases were reported to the Public Health Institute of the Republika Srpska (PHI RS), and the cumulative mortality rate was 547/100,000 [2].

Despite the enormous efforts placed into surveillance of COVID‐19 around the world, a significant proportion of cases remain undetected. Studies suggest that approximately 35%–40% of cases of COVID‐19 were asymptomatic [3, 4]. Asymptomatic infections play an important role in transmission of the SARS‐CoV‐2 and represent a major challenge in attempts to control the pandemic [5]. Additionally, overwhelmed laboratory diagnostics, epidemiological surveillance and contact tracing capacities, as well as declining population adherence to prescribed isolation measures and avoidance of testing, likely led to an under‐ascertainment of cases.

Results from seroepidemiological studies around the world indicate that COVID‐19 surveillance and reporting largely under‐estimates the true extent of infection and immunity, especially in low‐ and middle‐income countries (LMIC). Estimated seroprevalence‐to‐cumulative cases ratios in the second quarter of the 2021 ranged from 1.2:1 in high income countries of the WHO Region of the Americas to 183:1 in WHO Region of Africa, with the global median ratio of 17:1 [6]. In systematic review that compared SARS‐CoV‐2 seroprevalence and reported cumulative cases from 17 seroprevalence studies conducted before 12 August 2020, the estimated seroprevalence‐to‐cumulative cases ratio ranged from 0.56:1 to 717:1, and half of the studies reported ratio higher then 10:1 [7].

To understand the true extent of virus transmission, as well as to estimate under‐ascertainment of real number of cases, it is essential to perform the seroprevalence studies. These studies provide meaningful information which help us to understand the future course of the pandemic and to guide the public health response [6, 8].

The first COVID‐19 seroprevalence study in Republika Srpska, Bosnia and Herzegovina, was conducted as part of the WHO's UNITY initiative. The UNITY seroepidemiological investigation protocol (the SEROPREV protocol) is population‐based, age‐stratified protocol, and it provides a standard study design and laboratory approach to general population seroprevalence studies [6, 9, 10]. As of September 2021, SEROPREV has been implemented in 74 countries globally, including 51 LMICs [6], enabling further analysis of these comparable studies to answer the key questions about the progress of the pandemic globally.

The first population based seroprevalence study in Republika Srpska, Bosnia and Herzegovina, was conducted in December 2020, after the third wave of COVID‐19, before introduction of vaccination against COVID‐19, and it showed overall seroprevalence rate of 40.3%, with the 12:1 seroprevalence‐to‐cumulative cases ratio. Approximately 30% of seropositive individuals did not experience COVID‐19 symptoms. Subjects aged < 65 years were 2.06 times more likely to be seropositive than those aged ≥ 65 [11].

Vaccination against COVID‐19 in the Republika Srpska started in February 2021, almost 1 year after the first case of COVID‐19 was confirmed, and it was conducted according to the Plan for COVID‐19 vaccination in the Republika Srpska. Priority groups for vaccination were health care workers, residents and employees in nursing homes, people aged ≥ 65 and people with defined chronic illnesses and conditions.

In 1 year, from 12 February 2021, until 12 February 2022, a total of 719,224 doses of COVID‐19 vaccines were given to residences of the Republika Srpska, or 62,655 per 100.000 inhabitants (Official Report on COVID‐19 vaccination, PHI RS, unpublished). Apart from the first 2 months of the vaccination campaign, when the quantities of vaccines were limited, the availability and accessibility of vaccination were maintained continuously. Cold‐chain requirements for first available vaccines (−20°C for Gamaleya, +2°C–8°C for Sinopharm and −70°C for Pfizer vaccine) were immediately established in primary (PHI RS) and secondary (Regional PHI) vaccine supply stores. Three types of vaccines, inactivated, vector‐based and mRNA, from six different producers, were available. The vaccines were equally distributed throughout the Republika Srpska, and all residents could choose a preferred vaccine. Vaccination sites were established in PHI RS and in its five regional centres, 54 primary health centres and 11 hospitals.

Mass‐vaccination sites outside health facilities were established in several larger cities, with the aim of providing vaccination for all citizens rapidly, efficiently and equitably [12, 13].

According to the PHI RS report, the most commonly administered COVID‐19 vaccines were Sinopharm (38.1%), followed by Pfizer/BioNTech (27.1%), Sputnik V (26.6%), Astra Zeneca (5.2%), Sinovac (2.9%) and Moderna (0.1%). Vaccination coverage among citizens aged 16 years and older was approximately 40% as of 12 February 2022. The highest coverage was achieved in older people (62% of residents 65–79 years completed primary course with two doses), while it is negligible among young people and children (13.8% in 20–29 years, 3.9% in 15–19, 0.2% in 5–14).

In December 2021, the Faculties of Medicine at the University of Banja Luka and the University of Eastern Sarajevo, in collaboration with the PHI RS and the WHO, jointly launched the second round of a population‐based seroepidemiological study in the Republika Srpska, Bosnia and Herzegovina. Here, we present the key findings of this study.

## 2 Methods

### 2.1 Study Design and Participants

The second round of a COVID‐19 population‐based age‐stratified seroepidemiological study in the Republika Srpska, Bosnia and Herzegovina, which included testing for SARS‐CoV‐2 antibodies and an epidemiological survey, was conducted from 1 December 2021 to 28 February 2022 as a cross‐sectional study. The study unit was the household, and the sample size was stratified according to the population size of different municipalities.

The Republika Srpska, Bosnia and Herzegovina, is estimated to have a total of 408,825 households, with the total number of 1,147,902 inhabitants, and it is territorially organized in 10 cities and 54 municipalities [14]. The average household size is 2.91. A sample size of 3761 produces a two‐sided 95% confidence interval (CI) width equal to 0.035 when the sample seroprevalence is 0.40, according to the results of previous seroprevalence study. An additional 30% reserve sample would produce total sample size of 1686 households (4812 participants). The number of households to be recruited was determined by dividing the number of observation units by the average household size. The selection unit was the household, and the observation unit was the inhabitant.

The sample size was determined specifically for each city/municipality. Number of households was defined according to the number of inhabitants per city/municipality, taking into account the 30:70 ratio of urban and other households.

Each municipality has a local primary health care centre. Households were selected randomly from their data bases (lists of registered patients‐families at a family physician), and all residents of selected households were invited to participate. Where a selected household refused to participate, another household from the reserve list (an additional 30% of households selected randomly) was recruited instead. In addition, where a household refuses to give informed consent for the testing of small children, the adults were not sampled, and the next household was selected from the reserve list.

The total number of included participants was 4463 (1534 households) and the response rate was 92.75%. Children and adolescents were also included in the study, 11 children from the age group 3–6 years, 109 from the group 7–14 years and 137 from the group 15–18 years. All of them were unvaccinated.

The study excluded persons accommodated in student dormitories, boarding schools for children and youth with disabilities, nursing homes, prisons, monasteries and convents.

Selected participants were invited to visit their primary health care centres to complete the survey and undergo free serological testing.

### 2.2 Epidemiological Survey

Participants were given a questionnaire focused on COVID‐19 symptoms, contact with suspected or confirmed cases, understanding of disease prevention, and attitudes towards COVID‐19. The questionnaire consisted of 73 questions, divided into five sections: demographic characteristics; COVID‐19 symptom data; history of PCR positivity; COVID‐19 cases within the household; vaccination status (including dates of vaccination and type of vaccine received); risk perception of SARS‐CoV‐2 infection; compliance with the recommended prevention measures. The interviews were conducted by nurses from primary health centres, additionally trained according to the study protocol by epidemiologists from PHI RS, the Faculty of Medicine Banja Luka and the Faculty of Medicine Foča.

### 2.3 Detection of SARS‐CoV‐2 Antibodies

Blood specimens were collected from all participants (3 mL from children/5 mL from adults) and stored at 2°C–8°C until delivery to the laboratories. Blood specimens collection was performed by laboratory technicians of primary health care centres, additionally trained for this study by microbiologists from PHI RS, the Faculty of Medicine Banja Luka and the Faculty of Medicine Foča. Serum specimens were tested for the presence of total SARS‐CoV‐2 antibodies using the WANTAI Total Ab ELISA assay, which detects receptor‐binding domain (RBD) of SARS‐CoV‐2 spike protein, using a Euroimmun ELISA Analyzer I‐2P (EUROIMMUN MedizinischeLabordiagnostika AG, Lubeck, Germany). The cut‐off value set by the manufacturer for a positive test result is ≥ 1.1. The test has a sensitivity of 94.36% and a specificity of 100%.

All seropositive samples were divided in four groups: (1) participants with prior PCR confirmed SARS‐CoV‐2 infection, vaccinated (SARS‐CoV‐2+/vaccinated); (2) participants with no prior PCR confirmed SARS‐CoV‐2 infection, vaccinated (SARS‐CoV2−/vaccinated); (3) participants with prior PCR confirmed SARS‐CoV‐2 infection, unvaccinated (SARS‐CoV‐2+/unvaccinated); (4) participants with no prior PCR confirmed SARS‐CoV‐2 infection, unvaccinated (SARS‐CoV2−/unvaccinated). We randomly selected app. 300 samples from each group in order to conduct quantitative analysis using the Kantaro Quantitative SARS‐CoV‐2 IgG Antibody IVD Kit. An initial qualitative ELISA was performed against recombinant RBD, and positive samples tested by quantitative ELISA against full length SARS‐CoV‐2 Spike protein. The signal from unknown samples is compared to a calibration curve to generate a final result in arbitrary units per millilitre (AU/mL). If the calculated confidence interval value is ≥ 0.70, the sample was considered RBD positive and requires confirmation using the Spike ELISA. If it is < 0.7, the sample was negative and contained no detectable levels of antibodies to the RBD protein fragment of SARS‐CoV‐2 Spike protein. Samples falling below the limit of quantification (LoQ) of 3.20 AU/mL were considered negative.

### 2.4 Statistical Analysis

Descriptive statistics were calculated for baseline participant characteristics, symptoms, testing and vaccination status. Categorical data were presented as absolute numbers with frequencies. Numerical data were presented as means or medians. Kolmogorov–Smirnov and Shapiro–Vilkov test were used to test normal distribution. Seroprevalence was determined as a proportion of individuals with a positive test result for total antibodies. Baseline differences between groups were analysed using Pearson chi‐squared test for categorical variables. Kruskal Wallis H test was used to examine the difference between the three most used vaccine manufacturers by Kantaro titre values. Wilcoxon‐Mann–Whitney test is used for comparing the immunogenicity of different vaccines. All tests were two‐tailed. The *p* value of < 0.05 was considered statistically significant. Statistical analysis was performed using IBM SPSS Statistics 26 software. To adjust for age and gender distribution in sample, poststratification method is applied. Survey package in R for Windows was used to perform raking in order to obtain weight that will make correction for age and gender distribution. The proportion of genders (male, female) in population is 0.492, 0.508 and for age (0–19, 30–34, 35–49, 50–64 and 65+) is 0.180, 0.174, 0.212, 0.214 and 0.220, respectively.

### 2.5 Ethical Statement and Informed Consent

The study was approved by the Ethics Committee of the Faculty of Medicine Foča, University of East Sarajevo (Decision number: 01‐2‐8 dated 6 November 2020), and it is in accordance with the Research Ethics Review Committee of the World Health Organization. All the participants have signed the informed consent for their participation, while parents/caregivers signed consent for the participation of children.

## 3 Results

**FIGURE 1:**
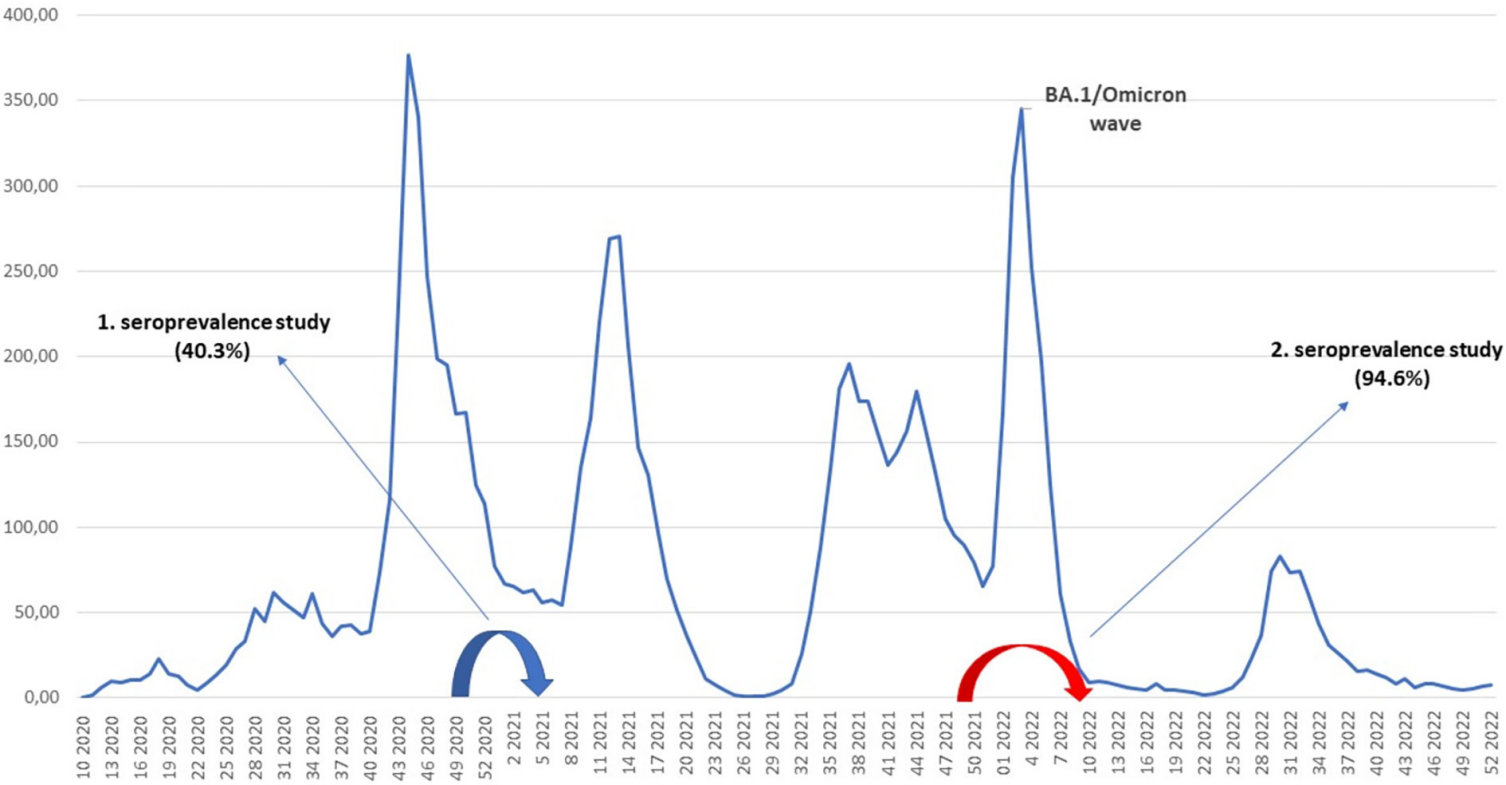
Weekly COVID‐19 incidence rates per 100.000 population and timing of seroprevalence studies.

The second round of COVID‐19 seroprevalence study in the Republika Srpska, Bosnia and Herzegovina, was conducted from 1 December 2021 to 28 February 2022, 1 year after the first seroprevalence study (Figure 1).

The study included 4463 participants, of whom 1723 (38.6%) were male and 2740 (61.4%) female. The mean age was 47.2 years, range 3–97 years (Table 1).

**TABLE 1:** Distribution of participants and seroprevalence according to gender and age groups.

	Total *N* (%)	Seropositive (*N*)	Prevalence % (95% CI)	*p* value	Weighted prevalence % (95% CI)^b^	*p* value
Gender						
Male	1723 (38.6%)	1605	93.2 (92.0–94.4)	< 0.001^a^	92.4 (91.2–93.4)	< 0.001^a^
Female	2740 (61.4%)	2617	95.5 (94.7–96.3)		95.2 (94.3–96.1)	
Age						
0–19	257 (5.8%)	234	91.0 (87.5–94.6)	0.014^a^	90.6 (88.5–92.5)	< 0.001^a^
20–34	741 (16.6%)	707	95.4 (93.9–96.9)		95.3 (93.6–96.6)	
35–49	1345 (30.1%)	1288	95.8 (94.7–96.8)		95.7 (94.2–96.8)	
50–64	1437 (32.2%)	1353	94.1 (92.9–95.4)		93.9 (92.3–95.3)	
≥ 65	683 (15.3%)	640	93.7 (91.9–95.5)		93.5 (91.8–94.9)	
Total	4463	4222	94.6 (93.9–95.3)		93.8 (93.1–94.5)	

^a^ Pearson chi square test.

^b^ Weighted prevalence—using poststratification method raking.

The overall seroprevalence in the Republika Srpska in this period was 94.6% (4222/4463), 95% CI = 93.9–95.3, while weighted prevalence was 93.8%, 95% CI = 93.1–94.5 (Table 1). There was no significant difference in seroprevalence between rural (94.7%, 95% CI = 93.4–96.0) and urban (94.7%, 95% CI = 93.9–95.6) areas. The seroprevalence among women was 93.2% and among men 95.5% (*p* = 0.001), and it ranged from 91.0% in participants 0–19 years to 95.8% in 35–49 years. Weighted prevalence among women was 92.4 (95% CI = 91.2–93.4) and among men 95.2% (95% CI = 94.3–96.1) (Table 1).

The seroprevalence was higher among employed (95.7%, 95% CI = 95.0–96.6) and retired participants (94.0%, 95% CI = 92.6–95.4), comparing to unemployed (92.0%, 95% CI = 89.8–94.2) and non‐working population—students, pupils and children (92.4%, 95% CI = 89.8–95.2) (*p* ≤ 0.001). Among various occupations, the highest seroprevalence was recorded in participants employed in tourism and service industry (98.1%, 95% CI = 95.6–100), education and higher education (98.0%, 95% CI = 96.4–99.6) and health sector (96.9%, 95% CI = 95.2–98.6); the lowest seroprevalence was recorded among participants working in agriculture (91.6% 95% CI = 87.2–96.2) and crafts (92.6%, 95% CI = 89.4–95.8), *p* ≤ 0.001 (Table 2).

**TABLE 2:** Distribution of participants and seroprevalence according to occupation.

	Seropositive		
	Total	*N*	% (95% CI)
Healthcare	387	375	96.9 (95.2–98.6)
Education	296	290	98.0 (96.4–99.6)
Industry	817	777	95.1 (93.6–96.6)
Agriculture	155	142	91.6 (87.2–96.2)
Trade and transport	482	466	96.7 (95.1–98.3)
Public sector and administration	480	461	96.0 (94.3–97.8)
Crafts	258	239	92.6 (89.4–95.8)
Tourism and service industry	108	106	98.1 (95.6–100)
Other	988	918	92.9 (91.3–94.5)
Total	3997	3774	95.0 (94.3–95.7)

In this study, 2100 (47.1%) participants experienced symptoms of COVID‐19 during 2021, and 96.4% (95% CI = 95.6–97.2) of them were seropositive. Of those who had no symptoms, 92.6% (95% CI = 91.8–94.1) were seropositive. The most frequent symptoms are shown in Table S1.

Of all participants, 183 (4.5%) were hospitalized for COVID‐19 symptoms at least once during pandemic, and 98.9% (95% CI = 97.4–100) of these were seropositive. Hospitalization period lasted 7–14 days in 45.6% of hospitalised cases, < 7 days in 29.6% and > 14 days in 24.9% cases.

Among all participants, 2734 (61.3%) received at least one dose of vaccine against COVID‐19; 4.6% of them (126 persons) received one dose, 67.8% (1854) two doses and 27.6% (754) three doses. The percentage of vaccinated participants increased by age: 8.6% in age group 0–19 years, 40.3% in 20–34 years, 61.4% in 35–48 years, 74.9% in 50–64 years and 83.2% in ≥ 65 years. The highest vaccination coverage was found among participants working in health sector (72.3%) and industry (70.3%) and the lowest in agriculture (56.7%) and education (57.3%).

Seroprevalence was significantly higher among vaccinated comparing to unvaccinated participants, regardless of COVID‐19 status (Table 3). Among 2734 vaccinated participants, 2674 (97.8%, 95% CI = 97.3–98.4) were seropositive and 60 (2.2%, 95% CI = 1.7–2.7) seronegative, and seroprevalence increases with the number of received doses. Among 1633 unvaccinated participants, 1462 (89.5%, 95% CI = 88.0–91.0) were seropositive and 171 (10.5%, 95% CI = 9.0–12.0) seronegative (χ^2^ = 139.802, *p* ≤ 0.001).

**TABLE 3:** Seroprevalence according to participants vaccination status and number of received doses.

	Total *N* (%)^b^	Positive serological test	% (95% CI)	*p* value	Weighted prevalence % (95% CI)^c^	*p* value
Number of received doses						
1	126 (4.6%)	120	95.2 (91.5–99.0)	0.002^a^	94.9 (89.3–97.7)	< 0.001^a^
2	1854 (67.8%)	1806	97.4 (96.7–98.1)		97.2 (96.3–97.9)	
3	754 (27.6%)	748	99.2 (98.6–99.8)		99.2 (98.4–99.7)	
Total vaccinated	2734	2674	97.8 (97.3–98.4)	< 0.001^a^	97.7 (97.0–98.2)	< 0.001^a^
Unvaccinated	1633	1462	89.5 (88.0–91.0)		89.2 (87.7–90.5)	

^a^ Pearson chi square test.

^b^ Percents from total vaccinated.

^c^ Weighted prevalence—using poststratification method raking.

Among 60 persons that did not have antibodies despite prior vaccination against COVID‐19 (2.2% of total number of vaccinated participants), as many as 48 (1.8% of total) received two doses, 6 (0.2%) received one and 6 (0.2%) received three doses of vaccines (Table 3). The majority (32/60) were vaccinated with Sinopharm vaccine.

Most of the participants who completed the primary vaccination course (two doses) were vaccinated with Sinopharm (35.3%), followed by Pfizer (30.7%), Sputnik (28.9%), Astra Zeneca (3.5%) and other vaccines (1.6%). The highest seroprevalence was seen in those who received two doses of the Pfizer/BioNTech vaccine (99.0%), followed by Sputnik (98.7%), Astra Zeneca (98.1%) and Sinopharm (95.1%), χ^2^ = 17.856, *p* ≤ 0.001.

The average time from the second dose to serological testing was 6.87 months for Pfizer/BioNTech recipients, 8.14 for Sputnik V and 7.24 for Sinopharm recipients.

In vaccinated participants, we compared seroprevalence among recipients of different vaccines and found statistically significant differences, χ^2^ = 20.99, *p* ≤ 0.001. Additionally, within the group of vaccinated, we compared seroprevalence among those who had symptoms of COVID‐19 in the last year with dose who had not, by vaccine type, in order to assess differences in immunity. Higher seroprevalence rates were observed in the vaccinated group who had experienced COVID‐19 symptoms, with the most pronounced increase among recipients of the Sinopharm vaccine (Table 4).

**TABLE 4:** Seroprevalence in vaccinated participants, with and without COVID‐19 symptoms in 2021, by vaccine type.

	COVID19 symptoms			
	No		Yes	
	Total	Positive *N* (%)	Total	Positive *N* (%)
Sputnik V	195	193 (99.0%)	231	228 (98.7%)
Sinopharm	233	213 (91.4%)	291	285 (97.9%)
Pfizer	206	203 (98.5%)	243	241 (99.2%)
Astra Zeneca	34	33 (97.1%)	18	18 (100%)
Others	12	11 (96.0%)	10	10 (100%)

Similarly, in participants who experienced COVID‐19 symptoms during 2021, the seroprevalence was significantly higher among those who received at least one dose of vaccine (98.8%, 95% CI = 98.2–99.4) comparing to unvaccinated participants (93.4%, 95% CI = 91.8–95.0), *p* ≤ 0.001 (Table 5).

**TABLE 5:** Seroprevalence in participants who experienced COVID‐19 symptoms during 2021, in relation to COVID‐19 vaccination status.

Vaccinated (1+ doses)	Total	Positive	% (95% CI)	*p* value	Weighted prevalence % (95% CI)^b^	*p* value
Yes	1185	1171	98.8 (98.2–99.4)	< 0.001^a^	98.9 (98.1–99.4)	< 0.001^a^
No	894	835	93.4 (91.8–95.0)		94.0 (92.4–95.3)	
Total	2079	2006	96.5 (95.7–97.3)		96.4 (95.6–97.2)	

^a^ Pearson chi square test.

^b^ Weighted prevalence—using poststratification method raking.

The anti‐S RBD IgG antibody titres were measured in 1273 participants, randomly selected into four groups based on vaccination status and history of PCR positivity, using a quantitative ELISA test. The antibody titres ranged from 0.8 to 62.9, with a mean of 53.9 and a median of 60.2 (SD = 16.7). Significant differences were observed between groups. The highest mean antibody level, 1094.4 AU/mL, was observed in those with hybrid immunity (SARS‐CoV‐2+/vaccinated), compared to 187.0 in the SARS‐CoV2−/vaccinated group, 120.3 in SARS‐CoV‐2+/unvaccinated and 71.4 in SARS‐CoV2−/unvaccinated group (*p* ≤ 0.001).

**FIGURE 2:**
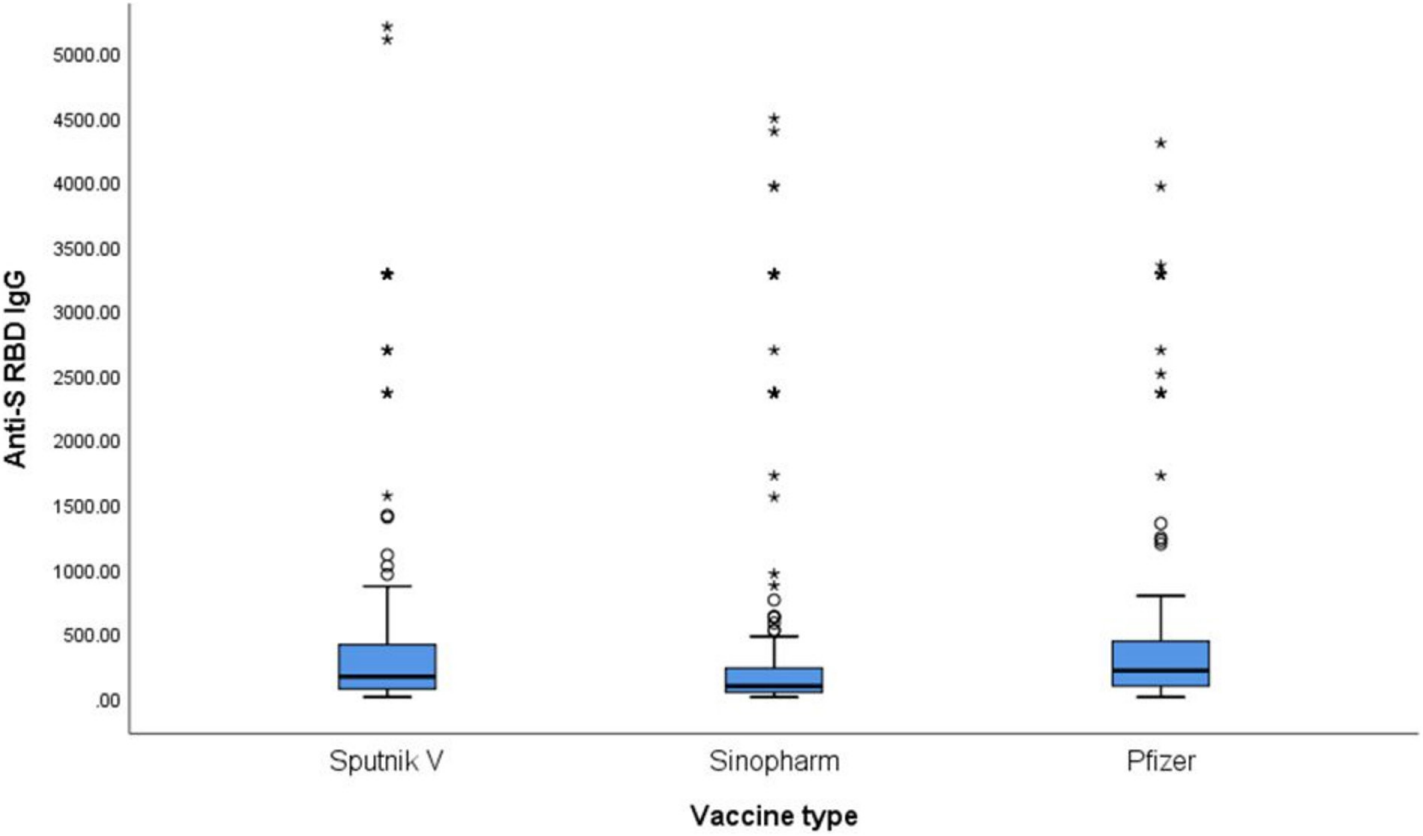
Differences in the distribution of anti‐S RBD IgG (Kantaro test) between recipients of different types of vaccines (subsamples).

Differences in antibody titres were observed among participants vaccinated (two doses) with different vaccines (Pfizer/BioNTech, Sputnik V or Sinopharm; *p* ≤ 0.001) (Table 6). After observing significance between pairs using the Wilcoxon test, the Mann–Whitney *U* test was applied. We found a significant difference in titres between recipients of the Pfizer/BioNTech and Sinopharm vaccines (*p* ≤ 0.001), as well as between Sputnik V and Sinopharm (*p* ≤ 0.001). Although the median titre was the highest among Pfizer recipients, there was no significant difference comparing to Sputnik V (*p* = 0.135; Table 6). When examining the distribution of IgG levels, we found a wider distribution in the Sputnik V group, with more positively skewed data, compared to the Pfizer group (Table 6, Figure 2).

**TABLE 6:** Comparative analyses and difference in values of anti‐S RBD IgG (Kantaro test) between recipients of different types of vaccines (subsamples).

No. of doses		*N*	Median (25th–75th percentile)	*p* value^a^	
				Sinopharm	Pfizer
1	Sputnik V	15	177.6 (88.8–414.1)	0.093	0.651
	Sinopharm	13	56.4 (30.7–177.2)		0.426
	Pfizer	8	188.1 (54.0–280.6)		
	Astra	1	83.6		
2	Sputnik V	189	159.6 (65.3–407.3)	< 0.001	0.135
	Sinopharm	212	86.8 (40.2–226.5)		< 0.001
	Pfizer	166	207.0 (85.9–433.7)		
	Astra	9	39.1 (13.2–169.1)		
3	Sputnik V	33	160.9 (72.6–322.6)	0.921	0.903
	Sinopharm	26	141.1 (49.3–723.1)		0.642
	Pfizer	11	130.1 (60.1–396.9)		
	Astra	2	68.8 (15.9–121.7)		
At least 1	Sputnik V	237	160.9 (65.4–401.4)	< 0.001	0.134
	Sinopharm	251	91.0 (39.0–246.9)		< 0.001
	Pfizer	185	206.7 (78.7–396.9)		
	Astra	12	61.4 (14.6–145.4)		

^a^ Mann–Whitney *U* test.

## 4 Discussion

The second population‐based seroprevalence study in the Republika Srpska, Bosnia and Herzegovina, showed 94.6% (95% CI = 93.9–95.3) SARS‐CoV‐2 seroprevalence between 1 December 2021 and 28 February 2022. The difference from 40.3% showed in the first study 1 year earlier (1 December 2020 to 15 January 2021) [11] indicates that further spread of SARS‐CoV‐2 and introduction of vaccination against COVID‐19 in the period between two studies resulted in 2.3‐fold increase in population seroprevalence. The highest increase was observed among participants aged ≥ 65 years, 3.7‐fold (from 26.0% to 93.7%), presumably due to the highest vaccination coverage in this group (Official Report on COVID‐19 vaccination in the Republika Srpska, PHI RS, unpublished).

According to the meta‐analysis of population‐based seroprevalence studies, the percentage of seropositive individuals increased in all regions during 2021, rising globally from 5.5% in June 2020 to 16% in February 2021, 45% in July 2021 and 67% by October 2021 [5]. This rise in seroprevalence in 2021 was also driven by an increased vaccination coverage, which was observed significantly in high‐income countries (HIC); 6% (January 2021) to 95% (August 2021) in America and 7% (January 2021) to 72% (August 2021) in Europe [5]. Since highly transmissible Omicron BA.1 sublineage of SARS‐CoV‐2 prevailed as the dominant variant globally at the beginning of 2022, producing a steep rise in the number of infections in many countries, both vaccine‐ and infection‐induced population seroprevalence levels were expected to increase substantially [15]. BA.1 caused huge peak of incidence rates in the Republika Srpska in January/February 2022, coinciding with the study period (Figure 1); thus it is expected that rapid rate of infection and trespassing of acquired immunity significantly increased seroprevalence and influenced results of the study.

In this study, we observed no significant differences in seroprevalence between rural and urban areas. The lower seroprevalence rates we found among women compared to men, as well as in the youngest (0–19 years) and the oldest age groups (≥ 65 years), which is in line with other studies results [6, 11, 16, 17]. In our first study, individuals aged ≥ 65 were 2.06 times less likely to be seropositive compared to individuals < 65 (*p* < 0.001), which can be explained by the social isolation and better compliance with recommended preventive measures [11]. However, in this study, the seroprevalence in the group ≥ 65 years became comparable to that in other age groups due to the highest increase, which we attribute to the highest vaccination coverage in population ≥ 65 years. This shows the importance of vaccination in achieving immunity among vulnerable older population, without exposure to potentially deadly virus.

Differences in seroprevalence were also observed by occupation type, with the highest values found among participants who work in the tourism and the service industry (98.1%), education and higher education (98.0%) and health care (96.9%). Since the beginning of the pandemic, health‐care workers are among professions with the highest risk of SARS‐CoV‐2 infection. In the Republika Srpska, Bosnia and Herzegovina, after the first 100 days of the pandemic, healthcare workers represented 6.8% of all confirmed cases [18]. A cross‐sectional study conducted in selected primary health care centres in the Republika Srpska between 19 March and 30 April 2021, found 69.5% seroprevalence among 1023 health‐care workers [19], significantly higher than the seroprevalence observed in the general population in the first population‐based study in January 2021. Vaccination coverage was higher among health care workers (72.3%) then in other professions, which likely contributed to their higher seroprevalence rate. In contrast, despite the low vaccination coverage of only 57.3% among participants who work in education, this group had one of the highest seroprevalence rates (98.0%).

The proportion of participants in this study vaccinated with at least one dose of COVID‐19 vaccine was 61.3%, with vaccination rates increasing with age, which is in line with the PHI RS data on vaccination coverage in the Republika Srpska. Despite the availability and accessibility of various COVID‐19 vaccines in the Republika Srpska, vaccine uptake was lower than expected, especially among younger individuals. However, PHI RS estimates that achieved vaccination coverage is noticeably higher than it is reported, having in mind that a significant number of residents were vaccinated in neighbouring countries, primarily in the Republic of Serbia. These vaccinations were not included in the coverage calculation, due to the lack of official data.

We consider vaccine hesitancy, influenced by perceived risks from the disease and perceived risks/benefits from the vaccination, as one of the most important factors contributing to insufficient vaccination acceptance in the Republika Srpska, similar to findings in various studies [20, 21]. According to a meta‐analysis conducted in May–June 2022, the global prevalence of COVID‐19 vaccination hesitancy was 25% [22]. The factors associated with a higher risk of COVID‐19 vaccination hesitancy in that study included: being a woman, aged ≤ 50 years old, single, unemployed, living in a household with five or more individuals, having an educational attainment lower than an undergraduate degree, having a non–healthcare‐related job and considering COVID‐19 vaccines to be unsafe. In contrast, factors associated with a lower risk of hasitancy included: living with children at home, maintaining physical distancing norms, testing for SARS‐CoV‐2 infection and having a history of influenza vaccination in the past few years [22]. Similarly, this study observed higher vaccination coverage among participants who had been PCR tested for SARS‐CoV‐2 (64.2%), compared to those who had never been tested (59.1%), participants ≥ 50 years compared to younger and among participants working in health care compared to those in other occupations.

Seroprevalence among vaccinated participants in this study was 97.8%, significantly higher compared to unvaccinated participants (89.5%). Numerous seroprevalence studies around the world showed significant association between the presence of antibodies and vaccination history [23, 24].

In our study, seroprevalence was even higher (98.8%) among participants with hybrid immunity, aquired trough both vaccination and prior infection, compared to those with only infection‐induced (93.4%) immunity (*p* ≤ 0.001). Additionally, the average level of anti‐S IgG antibodies among participants with hybrid immunity was 5.9 times higher than among those with vaccination‐induced immunity and 9.1 times higher than those with infection‐induced immunity. These findings correspond with numerous other studies, supporting the recommendation for vaccination, even for individuals with prior SARS‐CoV‐2 infection [25, 26, 27, 28].

According to the WHO recommendation from June 2022, hybrid immunity confers improved protection compared to infection‐induced immunity alone [15]. Therefore, achieving high primary vaccine series coverage in individuals in the highest and high‐risk groups, irrespective of their infection history, remains the foremost priority [17].

Observing different types of vaccines, individuals who received the Sinopharm vaccine in this study had significantly lower seroprevalence, 95.1%, in comparison with other available vaccines: Pfizer/BioNTech 99.0%, Sputnik 98.7% and Astra Zeneca 98.1% (*p* ≤ 0.001). A study from Jordan, between March and April 2021, recorded similar seroprevalence rate among Pfizer‐BioNTech recipients (99.3%), but lower among Sinopharm recipients (85.7%) [29]. A study from Egypt, conducted between January and June 2021, showed that 95.7% participants were tested seropositive (anti‐S antibodies) after receiving two doses of Sinopharm, similar as in our study [30].

Participants of our study who received two doses of Sinopharm and had never experienced symptoms of COVID‐19, were more often seronegative (8.6%) compared to recipients of other vaccines (Pfizer/BioNTech 1.5%, Sputnik 1.0% and Astra Zeneca 2.9%). However, Sinopharm recipients who experienced symptoms of COVID‐19 had significantly higher seroprevalence comparing to those who did not experience symptoms (97.9% vs. 91.4%), with seroprevalence levels similar to those observed in recipient of other vaccines. This finding could be important for assessing the protection and determining further vaccination schedules for recipients of inactivated vaccines.

We observed that 1.8% of participants who completed two‐dose vaccination course were seronegative. The average time between the second dose and testing in our study was > 6 months, which is issue of interest in the analysis of seroprevalence results. A study from the United Kingdom reported a 0.8% nonresponse rate: 1% among recipients of ChAdOx1 and 0.5% among recipients of BNT162b2, when measured ≥ 21 days after the second dose [31]. A study from Chile showed that 75% individuals who had received two doses remained seropositive at 180 days or more since their last dose; booster doses substantially increased seroprevalence after 180 days or more [32].

Analysing the quantitative tests results, we found significantly lower median levels of anti‐S antibodies in recipients of the Sinopharm vaccine, comparing to Pfizer/BioNTech and Sputnik V, consistent with other studies that found lower levels of antibodies after inactivated, compared to mRNA and vector‐based vaccines [33]. We did not observe significant differences between Pfizer/BioNTech and Sputnik V, although IgG levels where the highest in Pfizer group. Distribution patterns showed higher consistency of IgG levels among Pfizer recipients, while in the Sputnik V group, the mean was largely influenced by small number of individuals who experienced a significant increase in IgG titres (Table 6, Figure 2).

A systematic review conducted in 2022 suggests that all vaccines are effective in preventing COVID‐19. mRNA vaccines appear to be the most effective at preventing COVID‐19, while viral vector vaccines seem to be the most effective in reducing mortality [34].

Our study is a population‐based, age‐stratified seroepidemiological study that provides valuable data to enhance the understanding of SARS‐CoV‐2 transmission and the important role of vaccination in achieving population immunity. It contributes to the modelling or retrospective evaluation of transmission and vaccination dynamics in this region. The results of our study highlight the importance of COVID‐19 vaccination, irrespective of prior infection status. Considering that the majority of vaccinated persons in the Republika Srpska received inactivated vaccines, our findings support the recommendation for heterologous booster vaccination with different vaccine platforms to maximise the breadth of vaccine‐induced immunity [35].

Considering the overrepresentation of women in our study (61.4%) compared to the estimated population of the Republika Srpska (50.8%) and the higher average age in our sample (47.2 years comparing to 43.7 in the population) [36], we explored possibility that, despite the random selection of participants, the sample may not be fully representative of the entire population. We applied a poststratification method to adjust for age and gender distribution. The effect of poststratification was not substantial, with the weighted prevalence being 0.8 percentage points lower. Similarly, the weighted seroprevalence among women was 0.8, and among man 0.3 percentage points lower.

A limitation of the study is that we cannot distinguish between vaccine‐induced and infection‐induced antibodies. In addition, the fact that the study was initiated just before and continued through the BA.1 wave, could have affected the results due to several factors. The BA.1 wave was characterized by specific epidemiological factors, including increased transmissibility and potential immune escape, which could have resulted in a substantial increase in infections and seropositivity over the course of the study. Increased testing or awareness during a wave, could have lead to changes in population behaviour, potentially influencing who gets tested and who participated in the study, thus affecting its representativeness. The demographic profile of participants might also have differed during a wave, influenced by who is most affected or at risk during that period, potentially leading to biased results. We are unable to interpret this potential influences due to limitation in the available data.

## 5 Conclusions

Our findings reveal a 2.3‐fold increase in seroprevalence of SARS‐CoV‐2 antibodies over a one‐year period, driven by a combination of infection and vaccination. The most substantial rise (3.7‐fold) was observed in the age group ≥ 65 years, which achieved the highest vaccination coverage. Seroprevalence was significantly higher among vaccinated compared to unvaccinated participants, increased with the number of doses received and differed by the type of vaccine received. Additionally, antibody titres were higher among persons with hybrid immunity, as well as in those who received mRNA and vector‐based COVID‐19 vaccines, compared to recipients of inactivated vaccines.

Periodic population‐based seroprevalence studies are essential for evaluating surveillance systems, monitoring public compliance with recommended measures, and guiding vaccination policies as the pandemic progresses.

## Author Contributions

**Jela Aćimović:** conceptualization, data curation, formal analysis, writing – original draft. **Biljana Mijović:** conceptualization, data curation, formal analysis, investigation, methodology. **Srđan Mašić:** data curation, formal analysis. **Miroslav Petković:** formal analysis, methodology. **Dragana Puhalo Sladoje:** formal analysis, investigation. **Darija Knežević:** formal analysis, investigation. **Jelena Đaković Dević:** investigation, methodology, supervision. **Dragan Spaić:** formal analysis, investigation. **Jelena Vladičić‐Mašić:** validation, visualization. **Dejan Bokonjić:** conceptualization, funding acquisition, investigation, methodology, project administration, supervision. **Mirza Palo:** conceptualization, funding acquisition, resources, supervision. **Aisling Vaughan:** conceptualization, supervision, validation. **Richard Pebody:** methodology, validation. **Anthony Nardone:** methodology, project administration, resources. **Ranko Škrbić:** conceptualization, funding acquisition, methodology, project administration, resources, supervision.

## Ethics Statement

The study was approved by the Ethics Committee of the Faculty of Medicine Foča, University of East Sarajevo (Decision number: 01‐2‐8 dated 6 November 2020), and it is in accordance with the Research Ethics Review Committee of the World Health Organization.

## Conflicts of Interest

The authors declare no conflicts of interest.

### Peer Review

The peer review history for this article is available at https://www.webofscience.com/api/gateway/wos/peer‐review/10.1111/irv.70053.

## Supporting information


**Table S1** Supporting Information

## Data Availability

The data that support the findings of this study are available from the corresponding author upon reasonable request.
