# Supplementary material for: The Second Round of a Population‐Based Seroprevalence Study of Anti–SARS‐CoV‐2 Antibodies and COVID‐19 Vaccination Assessment in the Republika Srpska, Bosnia and Herzegovina
Source: Influenza Other Respir Viruses. 2025 Jan 16;19(1):e70053. doi: 10.1111/irv.70053 (PMC11739130; doi:10.1111/irv.70053)
Supplement: Supplementary file 1 — Table S1 Supporting Information [file IRV-19-e70053-s001.docx]

Table 1.

|  | Total | Seropositive n (%) |
| --- | --- | --- |
| Rhinorrhea |  |  |
| Yes | 2585 | 2469 (61.1) |
| No | 1566 | 1455 (36.0) |
| Unknown | 118 | 114 (2.8) |
| Cough |  |  |
| Yes | 2374 | 2275 (56.1) |
| No | 1782 | 1659 (40.9) |
| Unknown | 128 | 124 (3.1) |
| Sore throat |  |  |
| Yes | 2216 | 2120 (52.6) |
| No | 1904 | 1772 (43.9) |
| Unknown | 146 | 141 (3.5) |
| Headache |  |  |
| Yes | 2118 | 2033 (51.3) |
| No | 1950 | 1810 (45.7) |
| Unknown | 124 | 120 (3) |
| Fatigue |  |  |
| Yes | 2019 | 1940 (48.9) |
| No | 2070 | 1924 (48.5) |
| Unknown | 107 | 103 (2.6) |
| Body temp 38+ |  |  |
| Yes | 1662 | 1610 (40) |
| No | 2386 | 2214 (55) |
| Unknown | 211 | 203 (5) |
| Myalgia |  |  |
| Yes | 1708 | 1644 (41.5) |
| No | 2356 | 2195 (55.4) |
| Unknown | 127 | 123 (3.1) |
| Arthralgia |  |  |
| Yes | 1474 | 1416 (34) |
| No | 2555 | 2390 (57.3) |
| Unknown | 140 | 134 (3.2) |
| Anosmia |  |  |
| Yes | 1214 | 1191 (30.2) |
| No | 2822 | 2626 (66.5) |
| Unknown | 138 | 132 (3.3) |
| Ageusia |  |  |
| Yes | 1044 | 1021 (25.9) |
| No | 2982 | 2783 (70.6) |
| Unknown | 143 | 138 (3.5) |
| Dyspnea |  |  |
| Yes | 887 | 855 (21.6) |
| No | 3058 | 2873 (72.5) |
| Unknown | 245 | 235 (5.9) |
| Hospitalized due to any of the sympthoms |  |  |
| Yes | 183 | 181(4.7) |
| No | 3878 | 3363 (95.9) |

p<0.001
